# Improved gastrointestinal health for irritable bowel syndrome with metagenome-guided interventions

**DOI:** 10.1093/pcmedi/pbaa013

**Published:** 2020-04-29

**Authors:** Cem Meydan, Ebrahim Afshinnekoo, Nate Rickard, Guy Daniels, Laura Kunces, Theresa Hardy, Loukia Lili, Sarah Pesce, Paul Jacobson, Christopher E Mason, Joel Dudley, Bodi Zhang

**Affiliations:** Onegevity Health, 152 W 57th, New York, NY 10019, USA; Onegevity Health, 152 W 57th, New York, NY 10019, USA; Onegevity Health, 152 W 57th, New York, NY 10019, USA; Onegevity Health, 152 W 57th, New York, NY 10019, USA; Onegevity Health, 152 W 57th, New York, NY 10019, USA; Onegevity Health, 152 W 57th, New York, NY 10019, USA; Onegevity Health, 152 W 57th, New York, NY 10019, USA; Onegevity Health, 152 W 57th, New York, NY 10019, USA; Onegevity Health, 152 W 57th, New York, NY 10019, USA; Thorne Research, 152 W 57th, New York, NY 10019, USA; Onegevity Health, 152 W 57th, New York, NY 10019, USA; Onegevity Health, 152 W 57th, New York, NY 10019, USA; Onegevity Health, 152 W 57th, New York, NY 10019, USA; Thorne Research, 152 W 57th, New York, NY 10019, USA

**Keywords:** irritable bowel syndrome, microbiome, metagenomics

## Abstract

Irritable bowel syndrome (IBS) is the most prevalent functional gastrointestinal disorder worldwide, and the most common reason for referral to gastroenterology clinics. However, the pathophysiology is still not fully understood and consequently current management guidelines are very symptom-specific, leading to mixed results. Here we present a study of 88 individuals with IBS who had baseline sequencing of their gut microbiome (stool samples), received targeted interventions that included dietary, supplement, prebiotic/probiotic, and lifestyle recommendations for a 30-day period, and a follow-up sequencing of their gut microbiome. The study's objectives were to demonstrate unique metagenomic signatures across the IBS phenotypes and to validate whether metagenomic-guided interventions could lead to improvement of symptom scores in individuals with IBS. Enrolled subjects also completed a baseline and post-intervention questionnaire that assessed their symptom scores. The average symptom score of an individual with IBS at baseline was 160 and at the endpoint of the study the average symptom score of the cohort was 100.9. The mixed IBS subtype showed the most significant reduction in symptom scores across the different subtypes (average decrease by 102 points, *P* = 0.005). The metagenomics analysis reveals shifts in the microbiome post-intervention that have been cross-validated with the literature as being associated with improvement of IBS symptoms. Given the complex nature of IBS, further studies with larger sample sizes, more targeted analyses, and a broader population cohort are needed to explore these results further.

## Introduction

Irritable bowel syndrome (IBS) was first described over 150 years ago, yet it remains one of the most frustrating clinical challenges in the 21st century.^1,2^ The syndrome is a clinical diagnosis that belongs to the family of functional gastrointestinal (GI) disorders, a group of chronic conditions that are remarkably common and can generate significant healthcare, social, and economic burdens. IBS is the most prevalent functional GI disorder worldwide and the most common reason for referral to gastroenterology clinics;^3–6^ it impacts approximately 12% of the adult population worldwide, with a female (2:1) predominance.^3,7^ IBS is defined by recurrent episodes of abdominal pain associated with alternating bowel habits. The Rome IV criteria, widely regarded as the gold-standard for diagnosis, include recurrent abdominal pain at least 1 day per week for at least 3 months, and also defecation changes in at least two areas across: pain, frequency, and/or appearance.^8^ Currently, there is no clinical evidence to recommend the use of blood biomarkers for diagnosis.

Despite being described over a century ago, the exact etiology of the syndrome is unknown. The ‘biopsychosocial model’ is the prevailing theory, with several factors and mechanisms playing a role in the pathogenesis of the syndrome.^9^ These include altered gastrointestinal motility, visceral hypersensitivity, post-infectious reactivity, brain–gut interactions, alteration in fecal microbiome, bacterial overgrowth, food sensitivity, carbohydrate malabsorption, and intestinal inflammation, and all of these indicators have been studied as mechanisms involved in the pathogenesis of IBS.^10^ The syndrome has been further classified into subtypes based on the predominant bowel habit: IBS with predominant constipation (IBS-C), IBS with predominant diarrhea (IBS-D), mixed IBS (IBS-M), and un-subtyped IBS. Unfortunately, there is no definitive treatment for IBS and current management focuses on symptom alleviation. However, recent developments in the understanding of complex interaction between the gut, immune system, and nerve system offer novel therapeutic options for relief of both bowel movement-related symptoms and abdominal pain.^11–15^

One of these recent advances is the revolution of next-generation sequencing^16^ and its application to the microbiome. There has been a surge in studies seeking to understand the dynamics of these communities of microorganisms and the more complex role they play interacting with various organ systems, most notably the GI system, and impacting our health in a wide range of contexts.^17,18^ A common theory suggests that an imbalance in the gut microbiome leads to activation of the gut immune system and potential low-grade inflammation.^19–21^ Some key evidence supporting this hypothesis is increased risk of developing IBS after dysbiosis produced by acute gastroenteritis.^22^ Moreover, there have been several studies demonstrating differences in the composition of the gut microbiota of patients with IBS compared to healthy controls,^23–25^ unique signatures in patients with severe IBS,^26–28^ and alterations in the diversity and stability of the gut microbiome in patients with IBS.^29,30^ One study has also demonstrated that the mycobiome and community of fungal microorganisms is altered in patients with IBS and may be linked to severity of some symptoms.^31^ Overall, these studies demonstrate the potential role the microbiome will play in future diagnostics and therapeutics for patients with IBS.^18,20,32–34^ Of significance, however, most of these studies used 16S ribosomal RNA sequence, which is limited in the depth and scope in which it can characterize the microbiome of these patients, relative to the shotgun sequencing methods used in the present study.

Thus, we present a study of individuals with IBS using next-generation sequencing technologies to characterize their microbiome. The study's objectives were to demonstrate unique metagenomic signatures across the IBS phenotypes and to validate whether metagenomic-guided interventions could lead to improvement of symptom scores in individuals with IBS.

## Results

### Study design

We recruited and sent out microbiome collection kits to a total of 104 subjects to participate in our study, of whom 88 ultimately submitted their samples for baseline assessment. Sixty were individuals with IBS (21 constipation, 23 diarrhea, and 16 mixed phenotype), 10 were individuals with inflammatory bowel disease (IBD; 5 ulcerative colitis and 5 Crohn's disease), 13 individuals had other GI health-related symptoms (fungal overgrowth, small intestinal bacterial overgrowth, or leaky gut), and five were healthy controls. Participant ages ranged from 36 to 52 years, but the average age was 49 years. More women were enrolled in the study (60 female; 28 male), although this is also a consequence of the greater prevalence of IBS in women. People enrolled in the study from all across the United States with approximately half of the participants coming from the West, around 20% from the South and North East, and 7.5% from the Midwest.

**Figure 1.**
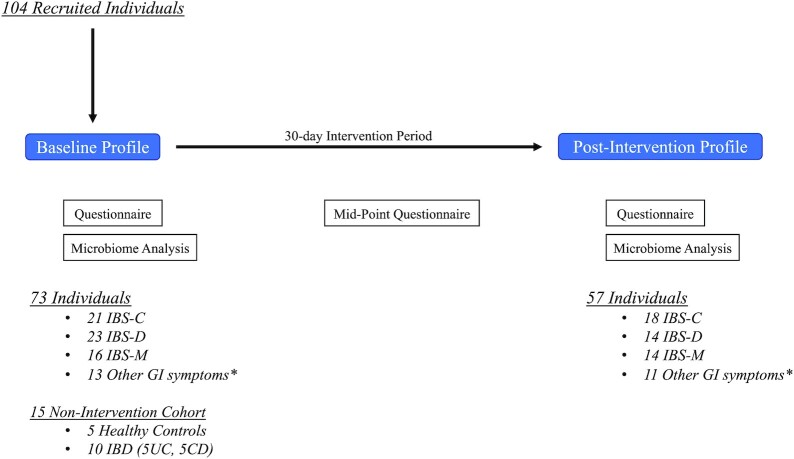
Study design. Individuals enrolled in the study begin with a baseline questionnaire and microbiome analysis before beginning their personalized intervention. After 15 days, an intermediate questionnaire is completed by all participants. After 30 days of intervention, at the endpoint of the trial there is a final questionnaire and microbiome analysis. Note the individuals in the ‘Other’ category self-reported other gut-related symptoms including leaky gut, gastritis, SIBO, etc.

Subjects’ stool samples were processed with shotgun sequencing metagenomics (see Methods) to 5.5 M reads per sample. The data analyzed from the baseline assessment were used to send a suggested intervention, individualized to each person based on Onegevity's nutrition-gut bacteria interaction database. The intervention includes prebiotics, probiotics, supplement, and dietary recommendations, and individuals were asked to follow these recommendations for a 30-day intervention period. Table S1 in the Supplementary Data highlights the key ingredients in the different interventions as well as support from clinical trials, *in vitro* studies, and review papers that report the impact these specific interventions have been shown to have on the gut microbiome. At the endpoint of this study, a post-intervention profile was conducted that, comparable to the baseline profile, included a questionnaire and microbiome analysis (Fig. 1). The study overall spanned approximately 3 months. Also, there was a mid-point questionnaire half-way (15 days) through the intervention period. Overall, 57 individuals followed up with a stool sample for microbiome analysis to complete their post-intervention profile. This cohort included 18 individuals with IBS-C, 14 with IBS-D, 14 with IBS-M, and 11 with other GI symptoms.

### Symptom score reduction

We developed a 9-point system of scoring symptom severity based on the Rome IV criteria to create a quantitative metric of the subjects’ GI status (see Methods). The symptom scores also helped to characterize all the enrolled subjects to their best respective cohort. As part of the baseline questionnaire individuals were asked to report if they had been diagnosed with any GI conditions. Some individuals self-reported as healthy despite having symptom scores in the 100s, thus the symptom scores helped to provide some objective measures in categorizing the participants based on their symptoms rather than depending solely on subjective, self-reported data.

**Figure 2.**
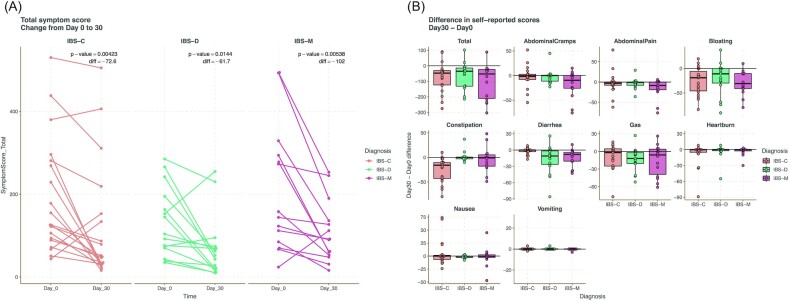
Symptom score reduction. (A) Trend lines of changes in calculated total symptom score over the course of the trial by different IBS phenotype. (B) Difference between day 30 and day 0 individual symptom scores highlighted by IBS phenotype.

The average symptom score of an individual with IBS was 160 (IBS-C: 180.1; IBS-D: 111.4; IBS-M: 203.6) compared to the average symptom score of 20.8 of our healthy controls. At the endpoint of the study the average symptom score of the IBS cohort was 100.9 (IBS-C: 119.6; IBS-D: 70.3; IBS-M: 108.2). Figure 2A shows the significant reduction in symptom scores across the IBS subtypes, with the greatest change seen in IBS-M (average decrease by 102 points, *P* = 0.005). Table S2 in the Supplementary Data shows all the metadata, symptom scores, and interventions for each individual enrolled in the study. The most significant changes were seen for constipation score of subjects with IBS-C (decrease of 25.7 points, *P* = 0.0003) and bloating in IBS-C (decrease of 26.4 points, *P* = 0.0006) and IBS-M (decrease of 29.1 points, *P* = 0.0007) (Fig. 2B). However, the ‘Other’ category of individuals with miscellaneous gastrointestinal conditions including small intestine bacterial overgrowth (SIBO), showed mixed results on their symptom scores and, on average, no change in total scores. This is because of an increase in bloating/gas symptoms for those subjects.

Dietary intervention alone did not lead to significant reductions in symptom scores. There were no significant differences between the reduction in symptom scores for previously gluten/dairy-free subjects compared to subjects who started the specialized diet after enrollment (Fig. S1 in the Supplementary Data). Subjects already following a dairy-free diet before enrolling in the study also showed a significant decrease in their total symptoms after using the supplements, comparable to subjects who became newly dairy-free during the trial. Scores decreased by an average of 91.8 in previously dairy-free (*P* = 0.0009) individuals, compared to a decrease of 71.4 in individuals who were not dairy-free before enrollment (*P* = 0.0004). Similar findings were seen with a gluten-free diet. There was a score decrease of 83.7 in previously gluten-free individuals (*P* = 0.002), compared to a decrease of 73.9 in others (*P*= 0.0004).

**Figure 3.**
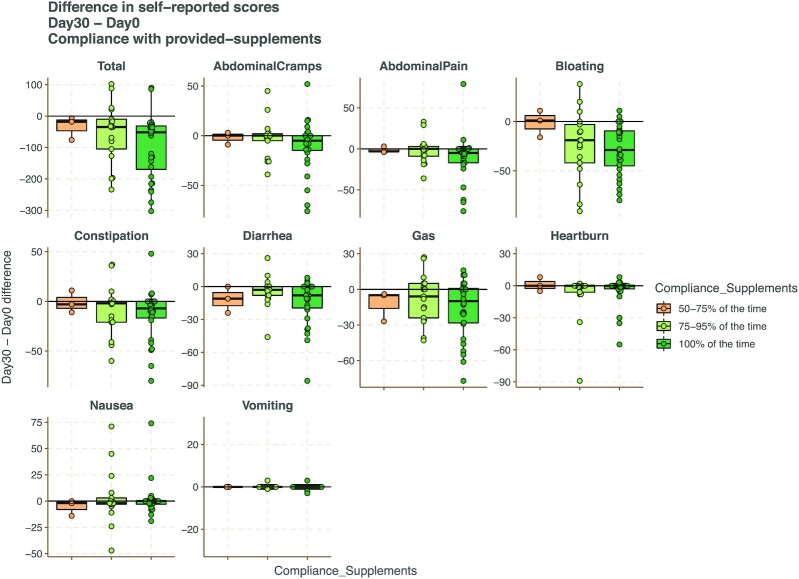
Impact of supplement compliance. Difference in self-reported symptoms scores based on compliance to supplements during the intervention period.

As the symptom improvement did not seem to be linked solely to dietary intervention, we then examined the GI alteration protocol type. Most probiotics, prebiotics, and supplements in the protocol worked about the same, although some exhibited better results for specific symptoms. For instance, Arabinex and *Bacillus coagulans* both showed a much higher decrease in constipation scores compared to other supplements (*P* = 0.0001, 26.8 IBS points and *P* = 0.0009, 23.3 better on average respectively), while Enteromend did not work well in constipation (*P* = 0.016, 16.8 points worse on average). However, Enteromend performed better than other mixtures for diarrhea symptoms (*P* = 0.00484, 15.2 points better on average), providing evidence that rational and customized therapies for patients can be implemented in this fashion. Moreover, the mid-point and endpoint questionnaires included compliance questions, and Fig. 3 shows that across the majority of symptoms 100% compliance to the supplement showed the greatest improvement in symptom scores.

### Metagenomic signatures separate phenotypes

Our results demonstrate that there may be metagenomics signatures in the taxonomic and functional pathway profiles of the gut microbiome in these patients that could be used to distinguish the subtypes in IBS. Table 1 highlights the top taxa enriched across the subjects. The predominant taxa identified across all subtypes were common gut microbiome species including *Bacterioides* species such as *B. vulgatus, B. fragilis, B. cellulosilyticus*, and *B. dorei*, as well as *Faecalibacterium prausnitzii, Akkermansia muciniphila, Eubacterium rectale*, and *Anaerostipes hadrus*. However, patients that were diagnosed as having IBS-C or had high constipation risk were found to have gut microbiome profiles with significantly increased levels of *Pseudomonas* and *Bacteroides thetaiotamicron*. Meanwhile they were found to have microbiomes depleted of *Paraprevotella* as well as significant signatures of *Fusobacterium nucleatum* and *Megamonas hypermegale*. Table S3 in the Supplementary Data shows the full microbiome taxa classification results across all the subjects.

**Table 1.** Top taxa across all subjects.^a^

Taxa	Avg Rel Ab	Total counts
*Bacteroides_vulgatus*	5.74719641	52 533 368
*Faecalibacterium_prausnitzii*	4.932946681	45 741 903
*Bacteroides_dorei*	2.918001597	27 083 646
*Eubacterium_rectale*	1.650263715	14 974 527
*Bacteroides_fragilis*	1.559081111	14 040 446
*Bacteroides_ovatus*	1.372481563	12 383 888
*Bacteroides_thetaiotaomicron*	1.371987424	13 052 862
*Bacteroides_cellulosilyticus*	1.122453283	10 776 118
*Eubacterium_siraeum*	0.969617082	8 880 775
*Lachnospiraceae_bacterium_GAM79*	0.913021632	8 543 597
*Ruminococcus_bicirculans*	0.800262527	7 319 595
*Anaerostipes_hadrus*	0.749038681	6 819 063
*Bacteroides_xylanisolvens*	0.747263722	6 715 923
*Roseburia_intestinalis*	0.670839292	6 138 100
*Bacteroides_caccae*	0.625557224	5 478 367
*Alistipes_finegoldii*	0.563761618	5 306 643
*Homo_sapiens*	0.514541639	4 333 695
*Akkermansia_muciniphila*	0.510487144	4 819 695
*Ruminococcus_sp_SR1/5*	0.472330465	4 309 006
*Alistipes_shahii*	0.455827333	4 169 931
*Eubacterium_eligens*	0.447791319	4 005 338

^a^This table features the top taxa as well as their average relative abundance and total counts across all samples. All of the taxa except for *Homo sapiens* were from the Bacteria domain. The most dominant genus was *Bacteroides* a common bacteria that makes up the gut microbiome and was found predominantly across all subjects regardless of IBS subtype.

**Figure 4.**
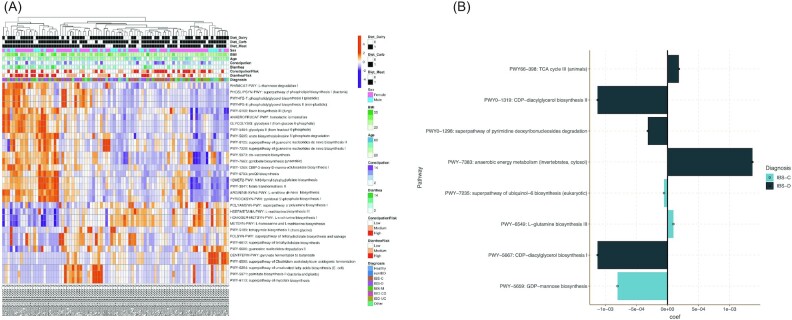
Biochemical pathway signatures and shifts. (A) This heatmap shows the functional pathways found across the baseline microbiome samples processed with different metadata fields from the self-reported questionnaire.. (B) Significant shifts in certain biochemical pathways post-intervention.

Figure 4A shows the profile of biochemical pathways found across the baseline cohort analysis. The top pathways found in diarrhea subject microbiomes were predominated by biosynthesis pathways of nucleotides and fatty acids, whereas constipation subjects had pathways more centered on sugar and amino acid metabolism. Figure 4B highlights the pathways that showed the most significant changes post-intervention. As expected, the pathways that showed the greatest shift were often linked with the predominant pathways (Fig. 4). For instance, IBS-D had increases in anaerobic metabolism and the citric acid cycle and decreases in nucleotide (CDP-diacylglycerol) biosynthesis, whereas IBS-C showed decreases in sugar (GDP-mannose) biosynthesis and amino acid (L-glutamine) biosynthesis. Table S4 in the Supplementary Data shows all the biochemical pathway results across all the subjects.

### Microbiome shifts pre- and post-intervention

**Figure 5.**
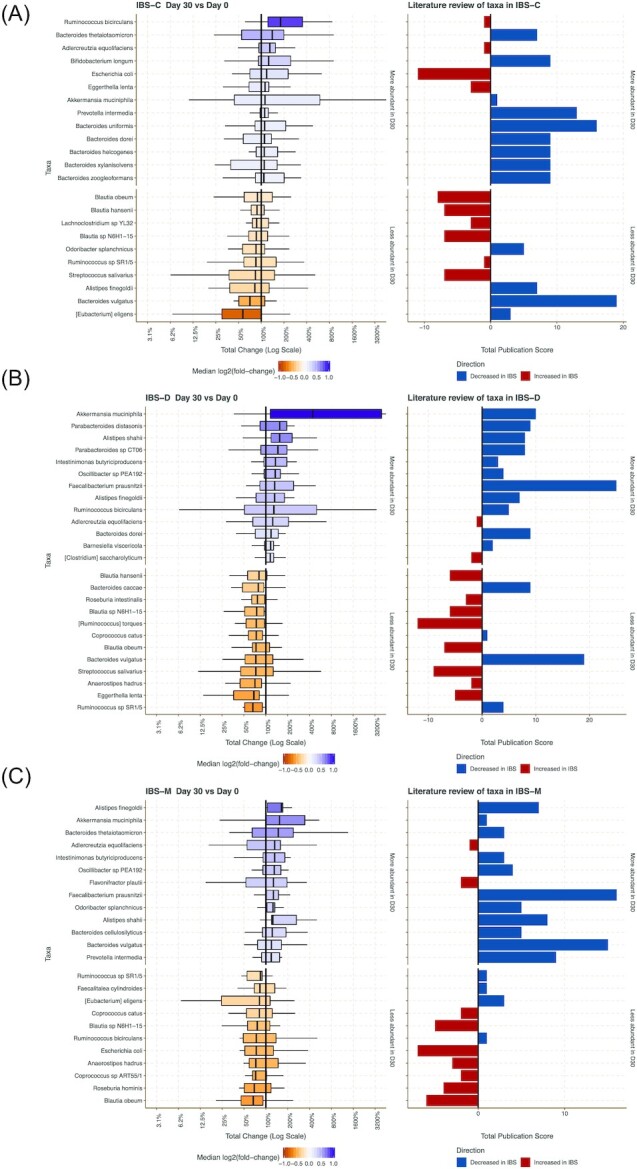
Post-intervention microbiome shifts. The top 20 species with the greatest log fold change across the IBS subtypes: IBS-C (A), IBS-D (B), IBS-M (C). Literature was reviewed to see which species have been shown to improve or worsen IBS symptoms and, based on this review, a literature score was calculated to compare with the results of the trial.

Figure 5 highlights the top species with the greatest total changes across all IBS subtypes post-intervention. Many of the species that increased post-intervention across all IBS subtypes were microorganisms commonly found in the gut microbiome, these include *Ruminococcus bicirculans, Akkermansia muciniphila*,and *Adlercreutzia equolifaciens*. The top taxa that decreased the most across all IBS subtypes included organisms associated with inflammation and dysbiosis including *Streptococcus, Erysipelotrichaceae*, and *Campylobacter jejuni*. Figure 5B summarizes literature review (Table S5 in the Supplementary Data) of recent IBS studies that have found specific taxa either linked with improvement or worsening of symptoms. Overall, the microbiome results from our trial comparing pre- and post-intervention effects demonstrate a consistent validation of the known microbial species linked with the improvement of symptom scores reported by the subjects.

### Human DNA marker

One of the advantages of whole genome shotgun sequencing over 16S amplicon sequencing is being able to examine sequence reads that align to eukaryotes, and in particular, the human host. We aligned all reads from each sample to the standard human genome reference and calculated the proportion of human reads present, which could come from lyzed or sloughed-off cells from the GI tract. Figure S2 in the Supplementary Data shows the relative abundance of human DNA across different subject disease categories, including data across our cohort's samples as well as other published IBD datasets. This result demonstrates that human DNA content from stool samples can be detected at significantly elevated levels in active flare ups for IBD, and to a lesser extent in IBS.

## Discussion

Overall, the results of our study suggest that metagenomic characterization including bacterial taxonomic and functional pathway profiling showed strong signatures that could be used to separate phenotypes. Literature review supports our findings of shifts in the microbiome and improvement in GI symptom score; however, further research is needed to determine any role biochemical pathways may play in pathophysiology and clinical manifestations of IBS. Moreover, the human DNA marker—especially as a measure for IBD or IBS has important implications and potential. There are millions of people affected by these disorders, and diagnostic tests (including blood tests and imaging studies) are only 80% accurate. Colonoscopy is the standard for definitive diagnosis, but it poses its own challenges including costs, inconvenience for the patient, and procedural risks. Moreover, patients with IBS have a higher probability of developing IBD during their life,^7^ further demonstrating the need for improved screening tools such as the human DNA marker, especially when integrated with other gut microbiome profiling.

The improvement in symptom score does seem to be driven partly by a supplement regimen in concert with dietary recommendations. The role of diet in management of IBS has been extensively reported.^35–37^ Moreover, studies have shown that probiotics are an effective therapeutic option for patients with IBS, with likely species-specific benefits for particular symptoms.^38-40^ A recent review and meta-analysis of over 50 randomized clinical trials found that particular combinations of probiotics, or specific species and strains, have beneficial effects on global IBS symptoms and abdominal pain, but did not draw definitive conclusions about their efficacy.^41^ The authors also reported that adverse events were no more common with probiotics or antibiotics across the studies. Moreover, patients with IBS often have comorbid psychological diagnoses including anxiety or depression. Recent studies suggest potentially similar pathophysiology in IBS and depression via the gut–brain axis,^42^ and other studies have found that probiotics improve psychological symptoms in healthy individuals.^43^

Nevertheless, further research is necessary to determine the optimal single- and multi-strain probiotics for IBS management. The most challenging aspect will be providing personalized recommendations as host characteristics will likely influence the efficacy of the probiotic on a patient's symptoms. Understanding and leveraging such predictors of response will be integral to optimizing the benefits of probiotics for patients with IBS. Tap *et al*., 2017,^26^ demonstrated a machine learning approach that allowed them to reduce the 16S amplicon sequence data complexity into a microbial signature for severe IBS consisting of 90 bacterial operational taxonomic units.^26^ They were not able to determine this signature using traditional and classical computational methods. Similarly, we used advances in artificial intelligence and machine learning approaches to develop a system of intervention recommendations that otherwise would be too difficult to develop manually. However, further data are needed to improve the system and demonstrate its true potential and efficacy.

Overall, our 30-day intervention period demonstrated an improvement in symptom scores, and this finding was further supported by the shifts in microbial composition between the baseline and endpoint microbiome analyses. However, the impact the microbiome has on health is complicated and will require further research, especially in complex syndromes such as IBS. There are complex relationships and interactions between the different species making up the gut microbiome, such as overgrowth of one species over another, as well as strain-level impacts on these relationships. For example, *E. coli* is a commensal microorganism but certain strains are pathogenic and can lead to worsening gut symptoms.

In the future, existing publications and literature on IBS, IBD, gut microbiome, diets, and pro/prebiotics could be combined with the medical history of the patient and longitudinal, multi-omic data on the patient, which would then be integrated together and used in a system to generate intervention recommendations and predictive metrics (e.g. DeepPatient).^44^ Such recommendations would include dietary changes, probiotics, prebiotics, and other lifestyle modifications. There are then three possible outcomes: the intervention does not improve symptoms, the intervention works but does not reach optimal results, or the intervention works well. The first situation would lead to an auto-correction loop that generates a second choice of interventions and recommendations, using additional medical information for improvement, and reviewing and stressing the importance of compliance. If the intervention works but does not reach optimal results, then the improvement loop would suggest stronger interventions with other products and also review and stress the importance of compliance. Finally, if the intervention works well, then the self-enforcement loop would reinforce the recommendation for future individuals with a similar profile.

Similar to the revolution sequencing technology had ushering in the era of precision medicine and transforming the current paradigm of diagnosing and treating cancer, metagenomic analysis is beginning to break new ground in the clinical realm and genome-guided care; in the present case, it is metagenome-guided care. For instance, short-chain fatty acids produced by the microbiome have been shown to regulate gut homeostasis and play a role in pathogenesis of type 2 diabetes and IBD, suggesting novel targets for therapeutics.^45,46^ Although there are still limitations to the full application of this technology and the field of microbiome research is still young, our understanding of its role in human physiology is continuing to grow and findings from this trial show clear potential, and evidence of gut microbiome signatures across patients with IBS—across the various subtypes—as well as how specific dietary interventions and the addition of targeted probiotics and prebiotics can lead to shifts in the microbiome and improve overall symptom profiles. However, this study is still limited in its sample size and scope, with a small control arm (five individuals) and non-diverse cohort (mostly white); thus, the conclusions we can draw from these results are constrained by what we see here. Especially with a syndrome like IBS, in which there are several variables and factors that impact the pathogenesis, with many confounding variables, these candidate microbiome profile changes could benefit from comprehensive long-term follow up, sampling, and analysis. Future studies should include larger sample sizes, more targeted analyses, and a broader population cohort to explore these results further.

## Methods

### Subjects

One-hundred and four participants were recruited, with 88 subjects ultimately enrolling in a controlled study to evaluate the efficacy and phenotypes of metagenomic based supplements/probiotics on participants’ gut health condition. The participants include patients with IBS (60), diarrhea (23), constipation (21), and mixed (16) subtypes, patients with other gastrointestinal issues (including IBD, SIBO, etc.; 23), and healthy controls (5).

### Regimen determination

Subjects with IBD received no recommended supplement intervention, healthy controls received no therapeutic intervention, and the subjects with IBS received unique three-product recommendations based on Onegevity's nutrition–gut bacteria interaction database, as described below. In addition, all subjects with IBS were recommended and guided to maintain a gluten-free and dairy-free diet for the duration of the study, and of note, a significant number of those entered the trial either gluten-free (39%), dairy-free (26%), or both (19%). Compliance with this dietary modification was deemed to be high.

### Questionnaire

A comprehensive questionnaire was completed prior to day 0 by all active subjects with IBS. It encompassed diet, food allergies/sensitivities, medications (prescribed or over-the-counter (OTC)/supplement use, diagnoses, relevant surgeries, and symptom scores. Various proprietary weights were given to determine the supplement regimen. For example, high diarrhea and constipation symptom scores were given significant influence. The questionnaire accounted for a portion of the supplement decision process.

### Symptom score calculation

The overall symptom score was calculated based on nine core symptoms: abdominal cramps, abdominal pain, bloating, constipation, diarrhea, gas, heartburn, nausea, and vomiting, where participants recorded the severity and frequency of each symptom on a scale of 1 (low) to 10 (high). The overall score was determined by taking the sum of the severity score and normalized by the frequency score across all nine symptoms

### Sample processing and analysis

Stool samples were collected using the Onegevity^TM^ GutBio^TM^ microbiome kit. Microbial DNA isolation from samples was carried out using an automated protocol and MoBio's PowerMag® (+ClearMag®) microbiome DNA isolation kit, on the KingFisherTM Flex instrument. Concentrations of extracted DNA from each sample were determined by Qubit measurement. An estimate of sample purity was also determined with spectrophotometry by measuring the A260/A280 and A260/230 absorbance ratios.

Next-generation sequencing libraries were prepared using the Nextera XT Library Prep from Illumina, in which the sample DNA is simultaneously enzymatically fragmented and tagged with primer sites for adapter/index addition. Sequencing adapters and indices were added during PCR amplification of the fragmented DNA. Fragment analysis on the Agilent Bioanalyzer was performed on a random selection of the samples to verify library size. DNA sequencing was done on the Illumina's NextSeq platform to produce 5–6 M reads per sample (150 × 150 read length).

As needed data was trimmed accordingly: any sequences matching at least three bases of the sequencing adapters (as defined by the library preparation kit) were trimmed to remove any adapters, any Ns (undetermined base calls) in the 5’ or 3’ of the reads were trimmed, any bases with sequence quality score <15 were trimmed, and any sequences of length <40 after these steps were removed from analysis. After quality control trimming steps, the samples were run through our bioinformatics pipeline for taxa classification and functional pathway analysis. Our bioinformatics pipeline includes krakenuniq^47,48^ with refseq database, which consists of bacteria, archaea, virus, fungi, human, as well as bracken2,^49^ MetaPhlAn2,^50,51^ and HUMAnN2.^52,53^

### Intervention types

A total of 10 options was available, with only three recommended to each subject based on their baseline microbiome, symptom score, and other criteria. Table S1 highlights the active ingredient across these interventions, which included supplements (glutamine, digestive enzymes, curcumin), probiotics (*B. coagulans, L. gasseri, B. longum, B. bifidum*), and prebiotics (arabinogalactan, pectin, and partially hydrolyzed guar gum). All recommended doses were kept uniform across all subjects. Supplement compliance was followed throughout the intervention period.

## IRB

The study protocol was approved by the Thorne Research committee on human research (TR-IRB #OH002). The study participants gave their written informed consent before enrolling in the trial.

## Supplementary Material

pbaa013_Supplemental_Files

## Data Availability

Data is available upon request.
